# Atypical Presentation of Appendicitis Leading to Exploratory Laparotomy

**DOI:** 10.7759/cureus.57848

**Published:** 2024-04-08

**Authors:** Bianca Patel, Mariam Nissan, Brian McMahon

**Affiliations:** 1 Pediatrics, Richmond University Medical Center, New York, USA

**Keywords:** laparotomy in appendicitis, unique marker of acute appendicitis, diagnosing appendicitis, clinical appendicitis, atypical appendicitis

## Abstract

Appendicitis typically presents with characteristic symptoms such as right lower quadrant abdominal pain, localizing to McBurney's point, facilitating diagnosis. Here, we report a case of a 19-year-old female who exhibited atypical manifestations, including lower abdominal pain and associated hematochezia. Despite inconclusive findings from imaging modalities and laboratory investigations, persistent pain prompted exploratory laparotomy, revealing appendicitis. This case highlights the diagnostic challenge posed by variant presentations of appendicitis, emphasizing the importance of clinical judgment and vigilance in surgical decision-making.

## Introduction

Appendicitis is common between ages 10 and 20 years, with an incidence of approximately 233 per 100,000 individuals [1]. Approximately 300,000 hospital admissions annually in the United States are attributed to appendicitis [2]. While appendicitis can be identified based on clinical symptoms such as right lower quadrant pain with tenderness, along with fever, nausea, and vomiting, there are several cases where the diagnosis is delayed due to atypical symptoms such as diminished pain sensation, hematochezia, abdominal distension, and signs of peritonitis such as rigidity and guarding [3,4]. A substantial risk factor for the development of appendicitis is a family history [5]. It is imperative to minimize prehospital delays in the diagnosis and management of appendicitis to mitigate the risk of adverse outcomes and complications, including sepsis, abscesses, perforation, and even mortality [6,7]. In this report, we describe a unique case of appendicitis characterized by atypical symptoms alongside unremarkable laboratory results and imaging findings to bring attention to a clinically skewed scenario that can make the initial diagnosis more complex.

## Case presentation

A 19-year-old female presented with a month-long duration of persistent abdominal pain localized to the lower quadrants, accompanied by bloody stools. The pain initially emanated from the lower quadrants but became concentrated in the right lower quadrant, corresponding to McBurney's point. Described as a burning sensation, the pain intermittently intensified into sharp shooting pains, particularly exacerbated by physical exertion and extending to the lower right back. Additionally, she reported loss of appetite and frequent nausea throughout the day, without any episodes of vomiting or diarrhea. Over-the-counter oral acetaminophen (Tylenol) was taken, resulting in limited alleviation of symptoms. The intensity of the pain was assessed at 7 out of 10. In addition to abdominal discomfort, she presented persistent bloody stools, and bowel movements failed to alleviate her abdominal pain. The patient's past medical and surgical records yielded no significant findings. Nonetheless, there is a familial predisposition to appendicitis. The patient reported no history of smoking, recreational drug use, alcohol consumption, or sexual activity. On the physical exam, the patient exhibited a cheerful demeanor, engaged in conversation, and demonstrated normal ambulation despite experiencing persistent pain. Abdominal assessment revealed tenderness in the lower quadrants, with rebound tenderness evident in the right lower quadrant, accompanied by a positive McBurney's sign.

Based on the clinical presentation and abdominal examination, the initial impression pointed towards a provisional diagnosis of acute appendicitis, with supratentorial elements included in the differential diagnosis due to this skewed presentation. Initially, due to the ongoing hematochezia, a colonoscopy was performed to rule out inflammatory bowel disease; however, the results revealed appendiceal orifice inflammation. As a result, an abdominal ultrasound was conducted to investigate potential appendicitis; however, the appendix was not visualized, thus precluding a definitive diagnosis of appendicitis. Following investigations, including a complete blood count, comprehensive metabolic panel, C-reactive protein, and lipase, all yielded normal results. In the pursuit of identifying the etiology of hematochezia, a Meckel's scan was performed; however, the findings were inconclusive.

**Figure 1:**
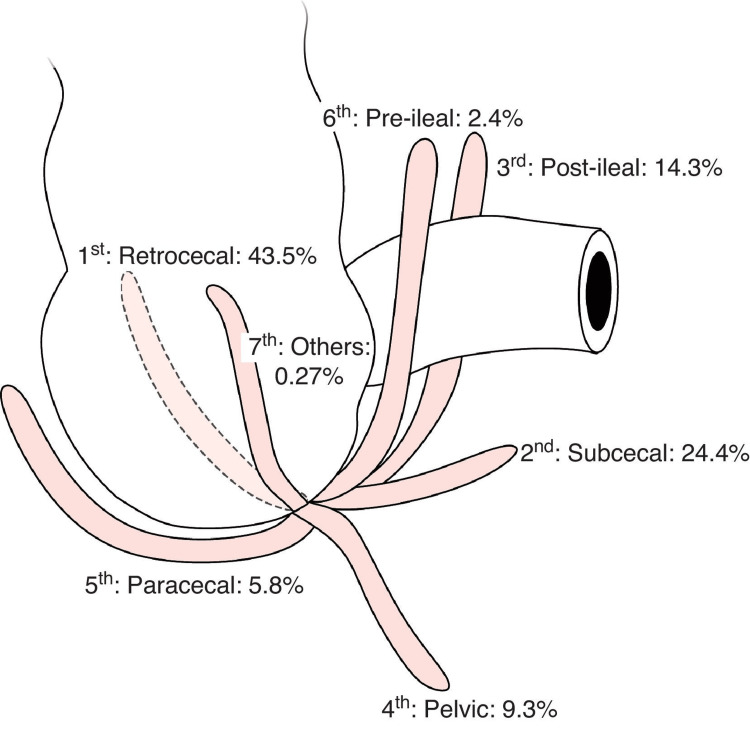
Positions of the vermiform appendix Image Credit: Souza et al. (2015) [8] This image is licensed under a Creative Commons Attribution-Non-Commercial-No Derivatives 4.0 International License: https://creativecommons.org/licenses/by-nc-nd/4.0/

Following the completion of noninvasive examinations, an exploratory laparotomy was deemed essential to investigate the atypical presentation further. Consequently, an appendectomy was performed after discovering an inflamed appendix twisted upon itself, located in a non-anatomical position within the pelvis, as illustrated by Souza et al. in Figure 1 [8]. Postoperatively, the patient exhibited a favorable response, with the cessation of constant pain, burning sensations, and hematochezia. One week following the procedure, the patient reported a pain score of 0 out of 10.

## Discussion

Acute appendicitis is characterized by classical symptoms including fever, right lower quadrant pain, and a positive McBurney’s sign. It is typically confirmed with elevated inflammatory markers and an ultrasound or CT scan [3,6]. In this case, the atypical manifestation of gastroenteritis symptoms, including persistent nausea, anorexia, and hematochezia, prompted the decision for an exploratory laparotomy and subsequent appendectomy [7,9,10]. It is crucial for clinicians to thoroughly explore the absence of typical symptoms of appendicitis to delay progression to more serious complications. Physical examination findings, such as rebound tenderness, should prompt further investigations, potentially necessitating procedures like an exploratory laparotomy, as illustrated in this case.

Neglecting atypical symptoms can lead to severe complications, including perforation, uncontrolled bleeding, or abscess formation, posing life-threatening risks [9,10]. Delays in diagnosis, visualization on imaging, and treatment of appendicitis can also lead to diffuse peritonitis, which may progress to significant morbidity and death [11,12]. Another consideration in cases where the appendix cannot be visualized on sonographic imaging is the possibility of non-anatomic positions, frequently observed in the posteromedial quadrant, sometimes positioned above the iliac crests or at depths beyond the capabilities of commonly utilized transducers with frequencies of at least 10 MHz, as demonstrated in this instance [11]. We emphasize the significance of a thorough investigation into atypical symptoms, which may indicate an appendix located in a non-anatomical position. In such cases, additional invasive diagnostic procedures, such as exploratory laparotomy, are deemed necessary to prevent life-threatening complications.

Atypical presentations of appendicitis can be misleading; nonetheless, it remains imperative for clinicians to prioritize the evaluation of these misleading symptoms. Further investigation, such as exploratory laparotomy, can prove crucial and potentially life-saving measures. Hence, laparoscopy serves as a valuable tool for both diagnostic and therapeutic purposes, particularly in cases involving anatomical variations.

## Conclusions

This was a 19-year-old female who presented with atypical symptoms such as diminished abdominal pain, hematochezia, rebound tenderness, and unremarkable labs and imaging, which necessitated an exploratory laparotomy and subsequent appendectomy following a thorough diagnostic evaluation. Post surgery, the patient’s pain subsided. It is crucial for clinicians to remain vigilant regarding the atypical symptoms and presentations of appendicitis, as failure to recognize these could precipitate life-threatening complications.
